# Thermal Degradation and Fire Properties of Fungal Mycelium and Mycelium - Biomass Composite Materials

**DOI:** 10.1038/s41598-018-36032-9

**Published:** 2018-12-04

**Authors:** Mitchell Jones, Tanmay Bhat, Everson Kandare, Ananya Thomas, Paul Joseph, Chaitali Dekiwadia, Richard Yuen, Sabu John, Jun Ma, Chun-Hui Wang

**Affiliations:** 1 School of Engineering, RMIT University, Melbourne, 3000 Australia; 2 Institute of Material Chemistry and Research, University of Vienna, Vienna, 1090 Austria; 3 School of Mechanical and Manufacturing Engineering, University of New South Wales, Sydney, 2052 Australia; 4 Institute of Sustainable Industries and Liveable Cities, Victoria University, Melbourne, 8001 Australia; 5 RMIT Microscopy and Microanalysis Facility, RMIT University, Melbourne, 3001 Australia; 6 Department of Architecture and Civil Engineering, City University of Hong Kong, Kowloon, Hong Kong; 7 School of Engineering, University of South Australia, Mawson Lakes, Adelaide, 5095 Australia

## Abstract

Mycelium and mycelium-biomass composites are emerging as new sustainable materials with useful flame-retardant potentials. Here we report a detailed characterisation of the thermal degradation and fire properties of fungal mycelium and mycelium-biomass composites. Measurements and analyses are carried out on key parameters such as decomposition temperatures, residual char, and gases evolved during pyrolysis. Pyrolysis flow combustion calorimetry (PCFC) evaluations reveal that the corresponding combustion propensity of mycelium is significantly lower compared to poly(methyl methacrylate) (PMMA) and polylactic acid (PLA), indicating that they are noticeably less prone to ignition and flaming combustion, and therefore safer to use. The hyphal diameters of mycelium decrease following pyrolysis. Cone calorimetry testing results show that the presence of mycelium has a positive influence on the fire reaction properties of wheat grains. This improvement is attributable to the relatively higher charring tendency of mycelium compared to wheat grain, which reduces the heat release rate (HRR) by acting as a thermal insulator and by limiting the supply of combustible gases to the flame front. The mycelium growth time has been found to yield no significant improvements in the fire properties of mycelium-wheat grain composites.

## Introduction

A new class of composite materials can be made by growing mycelium on various types of biomass, and the resultant composites have recently received significant attention due to their low density and biodegradable properties^1–7^. Mycelium is the vegetative growth of filamentous fungi, comprising of a network of micro-filaments with diameters ranging between 1 and 30 µm, depending on the type of species and growth environment^3^. Mycelium grows on organic substrates through apical tip expansion of hyphae from a spore or inoculum, under ambient conditions^8^. The hyphal colonies interact randomly through hyphal fusion (anastomosis) to form a fibre network structure^9^, which acts as a natural self-assembling glue to bind the substrates and form a composite material. The key incentives for the use of mycelium composites are their low cost, low environmental impact and carbon footprint, low density, reduced energy consumption and most importantly, their biodegradability^1,2,10^. Mycelium composites are currently being used in non-structural applications (e.g. packaging and insulation)^1,11^. Although the mechanical properties of mycelium composites based on biomass are inferior to those of conventional engineered (glass or carbon fibre) composites, advanced processing techniques have enabled their use in semi-structural applications (e.g. furniture, decking, etc.)^3–5,12^. Furthermore, the wide variety of substrates on which mycelium grows combined with advanced processing techniques provides manufacturers with new opportunities to customize the material to meet specific requirements (e.g. impact resistance, thermal and acoustic insulation, etc.)^2,7,13^.

Much of the potential applications of mycelium-based composites are intended for high fire prone environments (e.g. packaging and building insulation). However, very little is known about the thermal degradation and fire properties of mycelium and its composites. To meet the stringent fire safety regulations, it is imperative to characterize the flame-retardant properties of mycelium and mycelium composites. The heat, smoke and gases released by a burning composite can also make fire-fighting extremely hazardous and increase the likelihood of serious injury and death^14^. Large quantities of organic matter present in mycelium composites can act as a fuel source and may escalate the fire risk by shortening the ignition time, increasing heat release and other fire risk factors such as flame spread and smoke toxicity, although these are yet to be quantified. These issues and the effect of incubation period (growth time), which controls the relative mycelial mass, on the fire properties of mycelium composites need to be thoroughly quantified to enable wide practical applications.

Here, we investigate the thermal degradation properties and subsequent changes in the morphological and chemical structure of mycelium. A single mycelium species (*Trametes versicolor*) was selected in this study based on its growth kinetics^15^ and its availability. Parameters such as the onset of decomposition, residual char, evolved gases and heat release were measured. Changes to the physical structure, reduction in hyphal diameters and chemical composition following pyrolysis were investigated to gain an in-depth understanding of the thermal degradation and decomposition mechanisms. Also investigated is the effect of incubation period (growth time) for composites incorporating an organic substrate on their respective fire properties. The results from this first study provide new understanding and quantitative data on the fire safety of mycelium composites.

## Materials and Experimental Methodology

### Mycelium Composite Preparation

**Figure 1:**
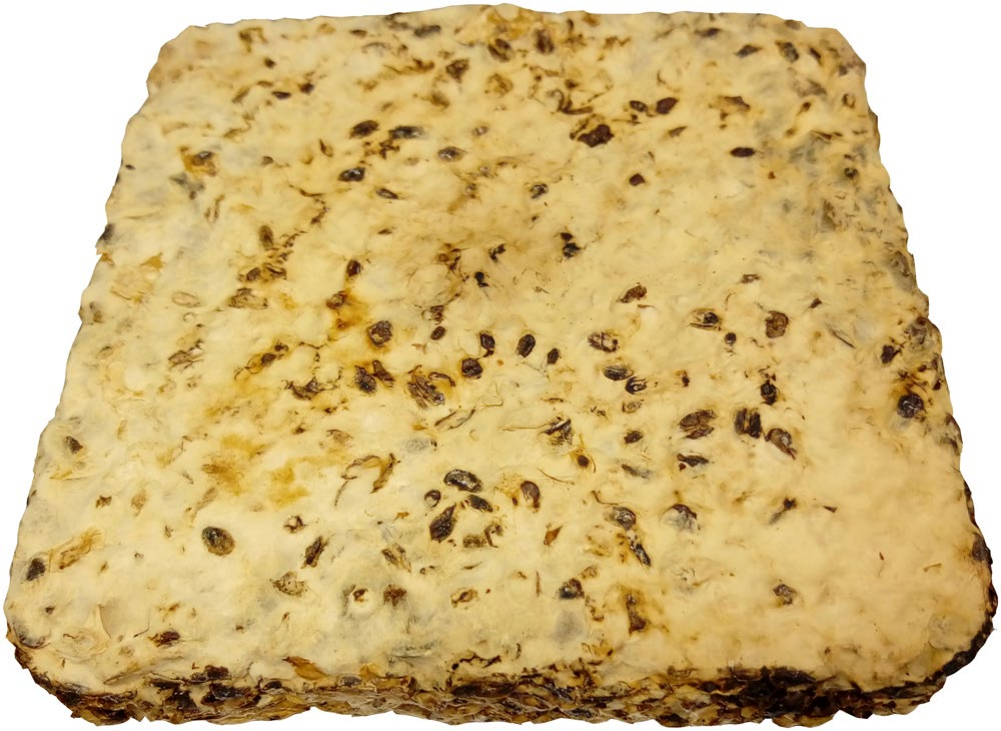
Representative mycelium (*T*. *versicolor*) – wheat grain composite grown for 18 d and dried at 50 °C for 48 h.

Fungal inoculum of the commonly used species (*T. versicolor*) was purchased from New Generation Mushroom Supplies Pty. Ltd. as mycelial mass growing on wheat grains. Wheat grains (supplied by E&A Salce Pty Ltd) were selected as a substrate material for their high nutritional value^16^ and to match the inoculum composition. The substrate (wheat grains) was soaked in Type 1 Milli-Q^®^ water for 48 h and sterilised (autoclaved at 121 °C and 103.4 kPa for 90 min) before use. A fixed amount (25 wt%) of fungal inoculum was then mixed with the substrate using a sterilised blender. Inoculum content lower than 25 wt% resulted in longer growth times and increased the risk of contamination by other competing microbial species. The blended mixture was then evenly distributed into sterile plastic moulds (100 × 100 mm) and incubated under standard atmospheric conditions for 6, 12 and 18 d. Following incubation, the specimens were dried at 50 °C for 48 h to dehydrate and denature the fungus. A representative mycelium-wheat grain composite is shown in Fig. 1.

### Coupled Thermogravimetric Analysis and Fourier Transform Infrared Spectroscopy (TGA-FTIR and TGA-GCMS)

To assess the mass loss and nature of the species evolved during thermal decomposition, measurements were conducted using a Pyris 1 TGA interfaced with a time-resolved FTIR (Perkin Elmer Frontier) and GCMS (Clarus SQ 8 S). A mycelial mass of approximately 10 mg was peeled from the composite, placed in an alumina crucible and heated from 25 °C to 600 °C at a heating rate of 30 °C/min under nitrogen atmosphere (50 ml/ min). All samples were inspected under an optical microscope prior to TGA measurements to ensure any bonded substrate (i.e. wheat grain particles) invisible to the naked eye were removed. The residue obtained following TGA was collected for further investigation into changes to the chemical composition.

The gases evolved during heating were piped (gas flow 50 mL/min) via a pressurized heated transfer line and analysed continuously by the FTIR equipped with a thermostated conventional gas cell. The infrared spectra were acquired in the 4000–600 cm^−1^ wavenumber range. At 300 °C, the evolved gas was automatically collected (*ca*. 80 µL) and injected into the gas chromatograph equipped with a standard non-polar fused silica capillary column which was then inter-phased to a mass spectrometer. Analyses of the average mass spectra calculated at the chromatographic peak middle height were carried out with NIST-MS Search Software.

### Scanning Electron Microscopy and Energy Dispersive X-Ray Spectroscopy (SEM-EDS)

SEM imaging and elemental analysis of mycelium before and after pyrolysis was performed using an FEI Quanta 200 Environmental Scanning Electron Microscope with an Oxford X-Max^N^ 20 Energy Dispersive X-ray Spectrometer attached. An accelerating voltage of 30 kV was used with a spot size of 5. The EDS spectra were analysed using the AZtecEnergy EDS software. An average spectrum was obtained based on individual spectra from 30 different sites.

### Transmission Electron Microscopy (TEM)

The *T. versicolor* hypha specimens were fixed in a 100 mM cacodylate buffer (pH 7.0) solution containing 2.5% glutaraldehyde, 2% paraformaldehyde followed by washing thrice with 100 mM cacodylate buffer. The specimens were post-fixed with 1% osmium tetroxide for 1.5 h followed by washing thrice with distilled water. The samples were gradually dehydrated with increasing gradients of 50% to 100% ethanol for 15–30 min each. Following dehydration, the samples were infiltrated twice with Spurr’s resin before polymerisation. The samples were post-polymerised at 60 °C for 48 h. Ultra-thin sections (~90 nm) were cut using the Leica Ultracut Ultramicrotome on carbon-formvar copper grids. The samples were post-stained with heavy metals and imaged using a JEOL-1010 transmission electron microscope (TEM) at 80 kV with the Gatan Microscopy Suite software (v 2.3) (Gatan Inc., Pleasanton, USA).

### X-ray Photon Spectroscopy (XPS)

The surface chemistry of the mycelium was assessed using a K-alpha X-ray Photoelectron Spectrometer (XPS) instrument (Thermo Fisher Scientific, USA) with a monochromated Aluminium Kα X-ray source. X-ray spot size was 30–400 μm in 5 μm steps. Scans spanned from 1400 to 0 eV binding energy. Peak analysis was performed by means of peak decomposition to fit a Gaussian function using the Thermo Scientific^TM^ Avantage Software (v 5.9902, b 06552). XPS spectra detail environment sensitive surface chemistry only as opposed to EDS which is a bulk analysis tool. As such XPS is considered to be less accurate than EDS for the elemental analysis of mycelial biomass and is included in the supplementary material for reference purposes only.

### Fire Reaction Testing

#### Pyrolysis Combustion Flow Calorimetry (PCFC)

The PCFC measurements were performed on mycelium using a Fire Testing Technology Ltd. (Gosport, UK) micro-scale combustion calorimeter according to ASTM D7309^17^. For each run, an accurately weighed (*ca*. 20 mg) sample was heated to *ca*. 900 °C at a heating rate of 1 °C/s, in a stream of nitrogen (80 cm^3^/min). The volatile thermal degradation products, thus obtained, were then mixed with a stream of pure oxygen (at a flow rate of 20 cm^3^/min) prior to entering a combustion chamber maintained at 900 °C. Each sample was run in duplicate. A more detailed description of the method including operating parameters is described by Cogen *et al*.^18^. The following quantities were measured: peak heat release rate (PHRR); temperature at peak heat release rate (TPHRR); total heat release (THR); heat release capacity (HRC) and percentages of the char residues^19^. These fire reaction properties of mycelium were compared against those of commonly used synthetic thermoplastic polymers PMMA and PLA for bench-marking purposes.

#### Cone Calorimetry

The flammability characteristics of the as-received wheat grains and mycelium-wheat grain composites grown for 6, 12 and 18 d were assessed using a three-cell cone calorimeter (Fire Testing Technology, UK) operated in the horizontal testing mode. The samples (100 mm × 100 mm) were exposed to a constant incident thermal heat flux of 50 kW/m^2^ in accordance with ISO 5660^20^ with the ignition event initiated by the pilot spark ignitor. The heat exposed surface was positioned 25 mm from the cone heater. The fire reaction parameters measured were time to ignition (TTI), heat release rate (HRR), mass loss and smoke density.

## Results and Discussion

### Thermal Degradation and Evolved Gas Analysis

**Figure 2:**
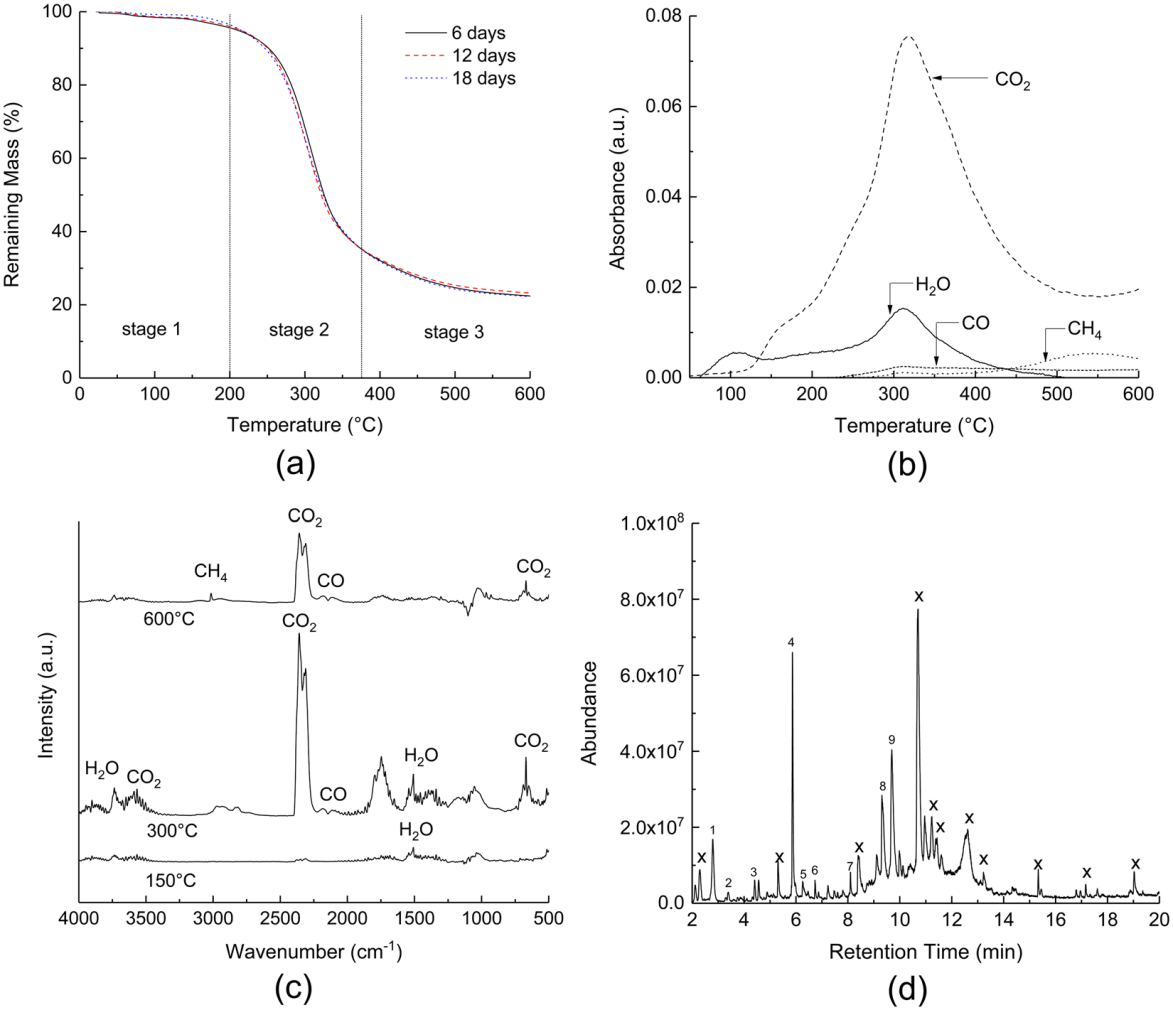
(**a**) TGA-mass loss temperature curve of mycelium grown for 6, 12 and 18 d between 25–600 °C under nitrogen (**b**) FTIR profiles of volatile products released expressed as a function of temperature (**c**) Offset Infrared spectra of the gases released at 150, 300 and 600 °C and (**d**) GC chromatogram of volatiles evolved at 300 °C respectively. Peaks marked with an ‘x’ symbol represent unidentified species.

Results of the mass loss and volatiles released during thermal decomposition measured by simultaneous thermogravimetric analysis and Fourier-transform infrared spectroscopy (TGA-FTIR) are shown in Fig. 2a. The TGA mass loss - temperature profiles exhibited three distinct stages with no significant differences between the thermal degradation characteristics of mycelium grown for 6, 12 and 18 d. The representative FTIR profiles of the major volatiles evolved were determined by relating the IR absorbance peaks to specific wave numbers (namely 3566 cm^−1^ for H_2_O, 3016 cm^−1^ for CH_4_, 2185 cm^−1^ for CO and 2359 cm^−1^ for CO_2_), and are shown in Fig. 2b. Monitoring the evolution of volatiles as a function of temperature, the FTIR analysis unveiled a complex degradation pattern characterized by the release of various flammable and non-flammable gaseous products.

The first degradation stage (25–200 °C) is attributed to the evaporation of free and chemically bonded water (H_2_O) leading to a mass loss of 5 wt% between 100 and 200 °C. The FTIR spectra (Fig. 2b) indicated mostly water being released up to 150 °C. A much larger second stage mass loss (*ca*. 70 wt%) occurs between 200–375 °C, presumably due to the decomposition of the organic constituents (e.g. amino acids, polysaccharides, chitin, etc.). FTIR analysis of the gases evolved showed that the mass loss in the second stage is accompanied by the release of a multi-component volatile mixture mainly composed of CO_2_ and H_2_O. The release of non-flammable gases such as CO_2_ and H_2_O is a critical finding since these gases may hinder flaming combustion by diluting the concentration of flammable gases evolved, thereby improving the fire performance. The FTIR profiles of CO_2_ and H_2_O reached their peak values at approximately 350 °C. Absorbance bands in the region of 2850 and 3030 cm^−1^ (Fig. 2c) revealed C-H stretching vibration due to the presence of volatile hydrocarbon moieties which are likely composed primarily of CH_4_. Moreover, absorbance peaks between 2000 and 2250 cm^−1^ show the presence of CO in the gaseous products, although the peak intensities are relatively low compared to those of CO_2_.

**Figure 3:**
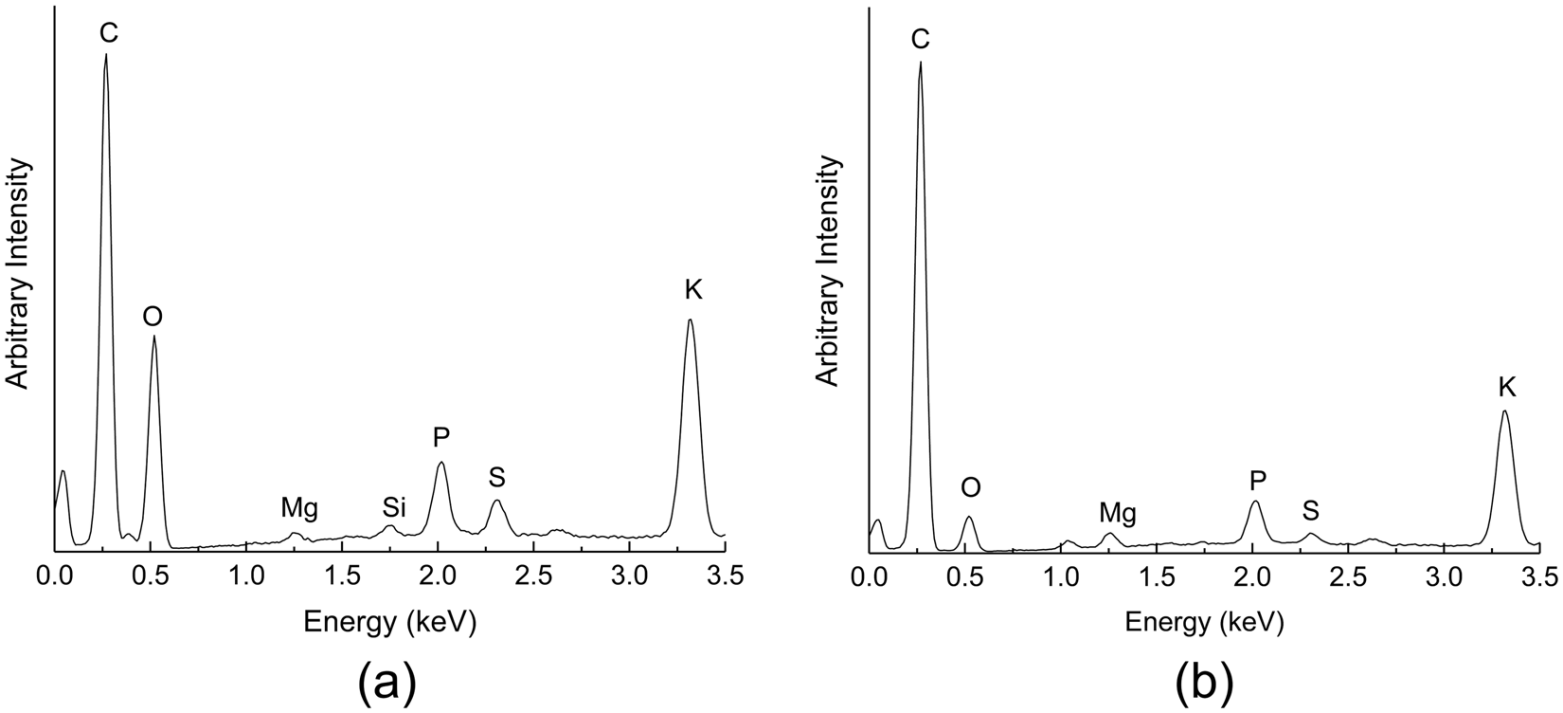
EDS spectra of mycelium (**a**) before and (**b**) after pyrolysis, respectively.

To further investigate secondary products during the thermal decomposition of mycelium, the evolved gases were also analysed using GCMS sampling of the volatiles evolved from the TGA at 300 °C. The gas chromatogram as shown in Fig. 2d, revealed the presence of several other organic species, many of which were aromatic in nature. The GCMS system was calibrated to identify gases between 45–450 atomic mass units (AMU) and hence could not identify low molecular weight gases such as CO_2_ and H_2_O. The wide variety and complex nature of compounds in mycelium made it difficult to identify all the gaseous products with high accuracy and hence, only products with a database match probability of greater than 60% are reported and listed in Table 1. Evidence for the presence of disulphide, dimethyl and heptasiloxane, hexadecamethyl- as volatile compounds released during decomposition of mycelium is supported through the detection of sulphur and silicon in the residual char via EDS measurements (Fig. 3a). No silicon was detected in the residue following decomposition, thereby suggesting its evolution in the gaseous phase. Likewise, a reduction to the carbon/sulphur peak intensity ratio in the residual char also suggests that sulphur derivatives may have been released as a gaseous product. The third and final decomposition stage (450–600 °C) involves further degradation of the primary residual char during which the chemical constituents have been completely reduced, resulting in the production of methane (CH_4_) and the consequent formation of a carbonaceous char residue. The carbon residue is expected to be fully amorphous due to the relatively low heating temperatures (<1000 °C without catalyst) in comparison to graphitizing temperature greater than 1400 °C^21^. The residual mass was found to be approximately 23 ± 0.6 wt% at 600 °C with a negligible drop at higher temperatures and is consistent with other studies performed on similar species of mycelium^2^. The relatively higher residue yield for mycelium compared to most thermoplastic^22–24^ and thermoset^25–28^ polymers implies a potentially lower tendency to form smoke and toxic volatiles during thermal decomposition/combustion, thereby suggesting improved fire safety for the former^29^.

**Table 1:** Anticipated volatiles released at 300 °C obtained from GCMS.

Peak Number	Compounds	Retention Time (min)
1	Furan, 3-methyl	2.79
2	Urea, methyl-	3.39
3	Disulphide, dimethyl	4.40
4	Furfural	5.86
5	2-Furanmethonal	6.25
6	4-Cyclopentene-1,3-dione	6.73
7	2-Furancarboxaldehyde, 5-methyl-	8.09
8	Diisooctyl phthalate	9.69
9	Heptasiloxane, hexadecamethyl-	9.97

The EDS measurements detected the presence of potassium and phosphorus in the residue, although their respective roles in the thermal decomposition of mycelium are still unclear. Phosphorus is widely used as a flame retardant in polymers and virtually any phosphorus compound can provide some degree of fire retardancy^14^. Since no phosphorus-based volatiles were detected, it is presumed that phosphorus was active within the condensed phase by promoting char formation through the production of phosphoric and polyphosphoric acids^14,25,30^.

### Morphological characterisation

**Figure 4:**
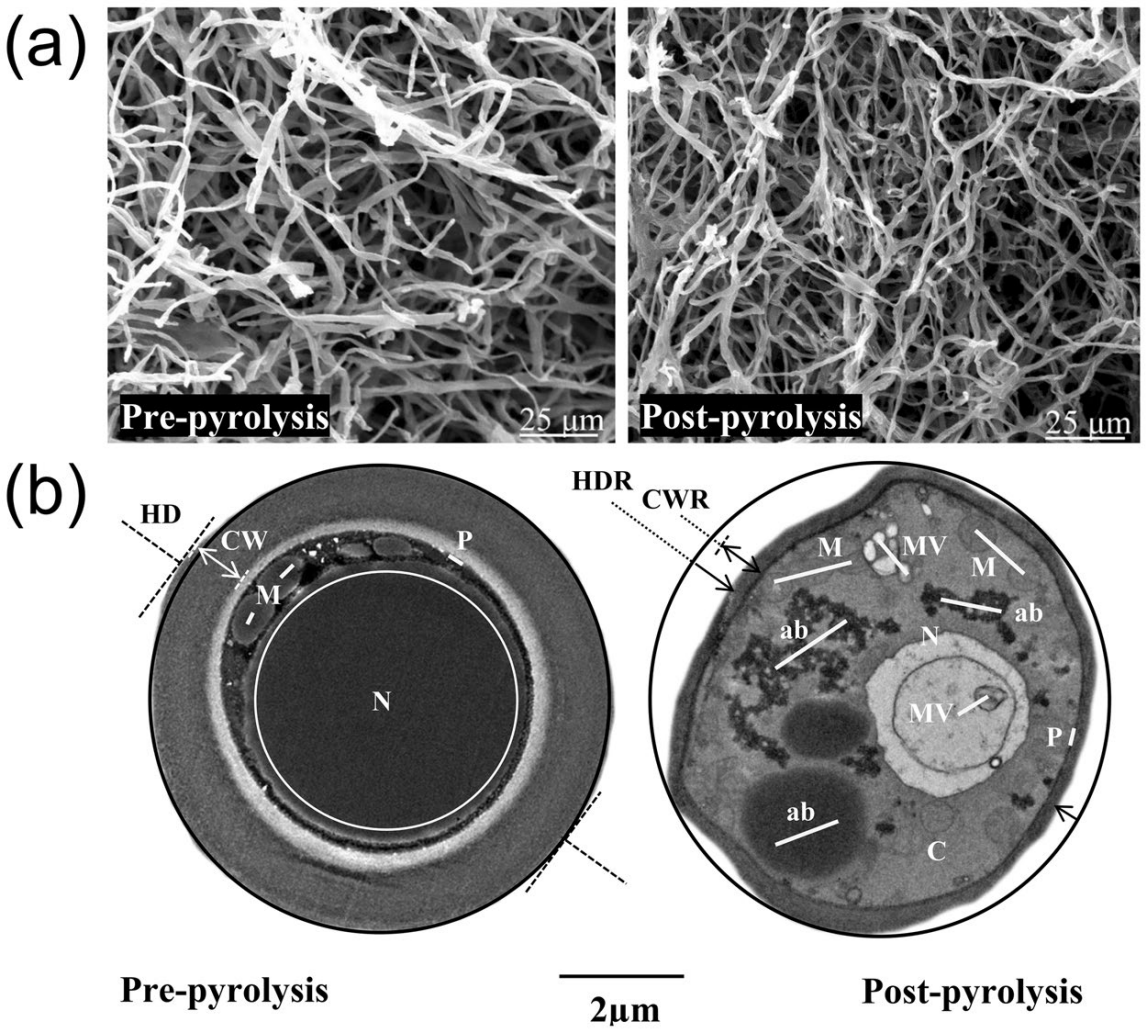
(**a**) Topographic SEM micrographs and (**b**) TEM transverse section micrographs detailing the ultrastructural, hyphal diameter and cell wall thickness changes in *T. versicolor* hypha pre- and post-pyrolysis (up to 600 °C in nitrogen). Abbreviations: ab, accumulation body; C, cytoplasm; CW, cell wall; CWR, cell wall reduction; HD, hyphal diameter; HDR, hyphal diameter reduction; M, mitochondria; MV, multivesicular; N, nucleus; P, plasmalemma.

Changes to the physical structure of mycelium after thermal decomposition were also investigated using the SEM and TEM. Micrographs were taken at various separate sites before and after pyrolysis and representative micrographs provided in Fig. 4. The hyphal diameters were measured using the Fiji distribution of ImageJ (v 1.51a) software.

As seen in Fig. 4, the fibrous network structure is retained after pyrolysis albeit with a substantial (10–30%) reduction in the hyphal diameter based on average hyphal diameter for 600 SEM hyphal diameter measurements and TEM sectional analysis after pyrolysis at 600 °C for 1 h. A 66% reduction in cell wall thickness was also identified based on TEM following pyrolysis. The retention of this fibrous structure is likely due to the presence of chitin in the cell walls of mycelium^31^. Chitin is a linear polymer of the acetylated amino sugar N-acetylglucosamine that forms microfibrillar arrangements in living organisms^32^. Chitin has a tensile strength greater than those of carbon fibres and steel^33^ and possesses excellent thermal stability and flame-retardant properties^34,35^.

This is an important finding which shows that the hyphal diameter can be tuned using thermal treatment. Species with a hyphal diameter of 1–3 μm can be pyrolysed to produce micro/nano sized carbon fibres that can then be used to toughen polymer-matrix composites for structural and non-structural engineering applications.

## Combustion Properties of Mycelium Composites

### Pyrolysis Flow Combustion Calorimetry

In investigating the fire reaction properties of pure mycelium, PCFC was chosen over cone calorimetry since it provides a rapid screening technique that requires only a few milligrams of a solid material and provides a direct comparison on the impact of replacing a synthetic polymer with mycelium as a binder in a composite material. In addition, the influence of sample morphology, thickness, heat losses through the sample-holder, etc. of conventional cone measurements are eliminated. A summary of properties measured using PCFC is shown in Table 2.

**Table 2:** Fire reaction properties of mycelium and other thermoplastic polymers measured using PCFC.

Sample	Temp to PHRR (°C)	pHRR (W/g)	THR (kJ/g)	Heat Release Capacity (J/gk)	Char yield (wt %)
Mycelium	300 ± 1	67 ± 2	6.8 ± 0.1	70 ± 1	23 ± 1
PMMA	399 ± 2	446 ± 6	24.6 ± 0.2	439 ± 6	0
PLA^23,37^	385	375	17.8	489	0.6

In addition to mass loss and evolved gases, the thermal degradation of mycelium is also accompanied by energy release in the form of heat. The heat release rate of a burning material is commonly accepted as the most important fire reaction property since it strongly influences factors such as flame spread, secondary ignitions, smoke generation, etc.^36^.

**Figure 5:**
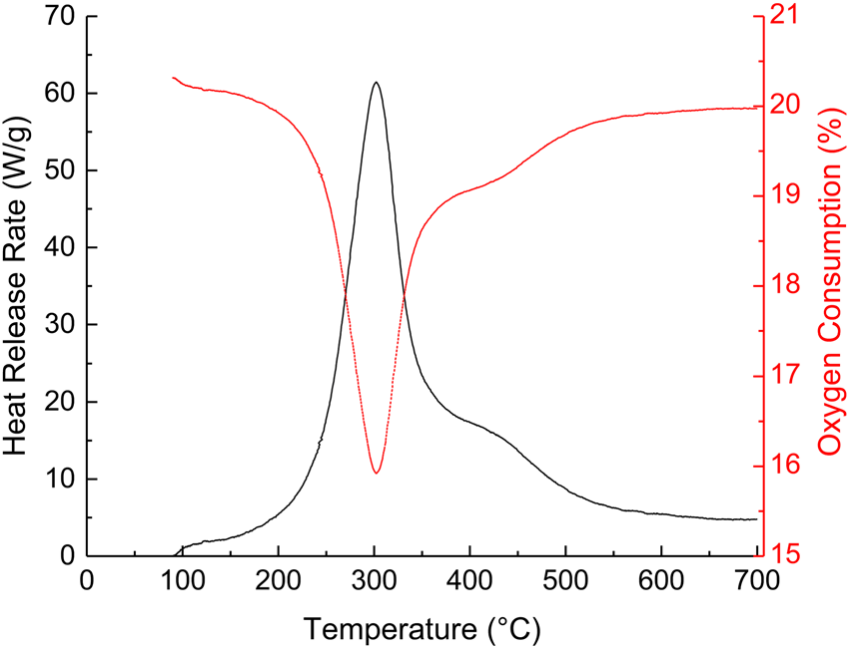
Effect of increasing temperature on the heat release rates and oxygen consumption of mycelium.

The heat release rate profile of mycelium fluctuates considerably with increasing temperature due to various chemical and thermal events that occur simultaneously during thermal degradation. Figure 5 shows an initial induction period (up to 100 °C) during which the material does not release any heat (indicative of a non-combustion phase). The mass loss (Fig. 2a) during this stage is independent of oxygen consumption (Fig. 5), and thus can be attributed fully to the loss of physically adsorbed water. The increase in heat release between 100 and 200 °C can be attributed to the release of flammable low molecular weight volatiles. Mycelium starts to decompose at approximately 225 °C. This event is accompanied by a rapid rise in the HRR spectrum reaching a peak HRR value at 300 °C, after which the heat release rate gradually decreases with temperature. The oxygen consumption reached a maximum value (*ca*. from 21 to 16 vol %) at the same time/temperature as the peak HRR (*ca*. 300 °C). The char yield obtained from PCFC tests was consistent with that from the TGA at approximately 23 wt%.

The fire reaction properties of mycelium are compared against commercially available polymers such as PMMA and PLA. The values for mycelium (PHHR = 67 W/g; THR = 6.8 kJ/g) are significantly lower when compared to both PMMA (PHHR = 446 W/g; THR = 24.6 kJ/g) and PLA (PHHR = 375 W/g; THR = 21.5 kJ/g)^23,37^, indicating that this material is less combustible and has improved fire safety over PMMA and PLA. The average heat release capacity of mycelium (69.5 J/gK) is also significantly lower than that of PMMA (439 J/gK) and PLA (489 J/gK)^23,37^, indicating superior resistance to flaming combustion. The improved flaming combustion resistance may be attributed to the higher residual char produced of mycelium (23 wt %) in comparison to PMMA (0 wt %) and PLA (0.6 wt %)^23,37^. The presence of char inhibits oxygen migration at the solid/gas phase interface thereby limiting the flaming combustion process.

### Effect of Incubation Period on Fire Reaction Properties of Composites

**Figure 6:**
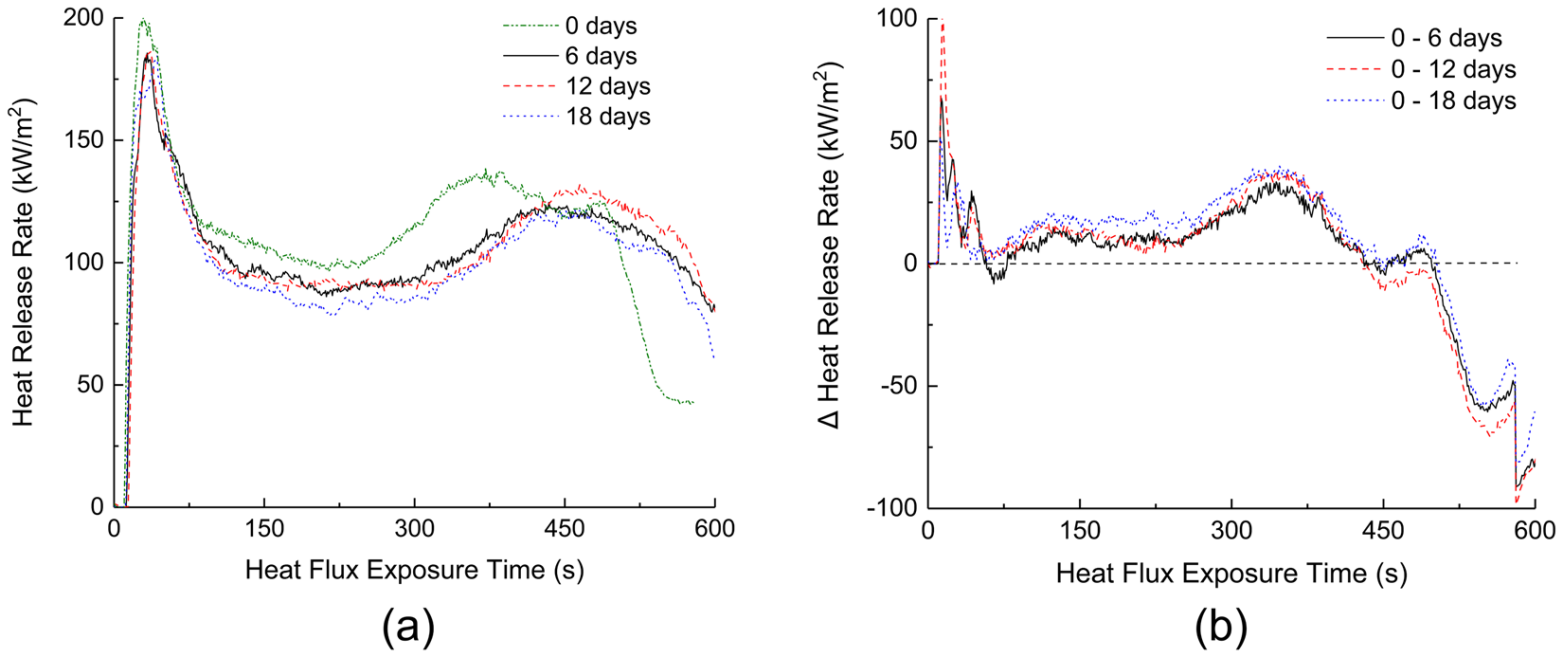
(**a**) Heat release rate data for composites grown for 0, 6, 12 and 18 d at an incident heat flux of 50 kW/m^2^ and (**b**) calculated Δ heat release rates, respectively.

In addition to pure mycelium, the fire reaction properties of composites made from mycelium and wheat grain were measured experimentally using a cone calorimeter at an incident heat flux of 50 kW/m^2^, with temperatures ranging from 600 to 700 °C^38–40^. The composites were grown for 6, 12 and 18 d to ascertain the differences, if any, in their fire reaction properties associated with growth time. The fire reaction properties were compared against those of the as-received wheat grains without mycelium (i.e. growth time of 0 d). As shown in Fig. 6a, the HRR profiles for all four samples are similar, with a rapid increase following ignition to reach an initial peak value. However, the peak heat release rate, which is considered a critical property controlling the maximum temperature and flame spread rate,^14,36^ was marginally higher (*ca*. 10%) for the 0-d sample (200 kW/m^2^) relative to that of composites grown for 6, 12 and 18 d (180 kW/m^2^). This observation suggests that the addition of mycelium results in marginal improvements to the fire reaction properties of the wheat grain substrate. The dissimilarity in flaming-combustion intensities of the 0, 6, 12 and 18-d composites is clearly depicted in Fig. 6b, wherein the ΔHRR-time profile is shown. The ΔHRR values were obtained by subtracting the HRR data measured for 0-d samples from those measured for 6, 12 and 18 d (i.e. HRR_0 d_ minus HRR_6, 12, 18 d_). The presence of the mycelium in the composite reduced the flaming intensity of the 6, 12 and 18-d samples during the initial thermal exposure (e.g. first 350 s) as revealed by the positive ΔHRR values over this period.

**Figure 7:**
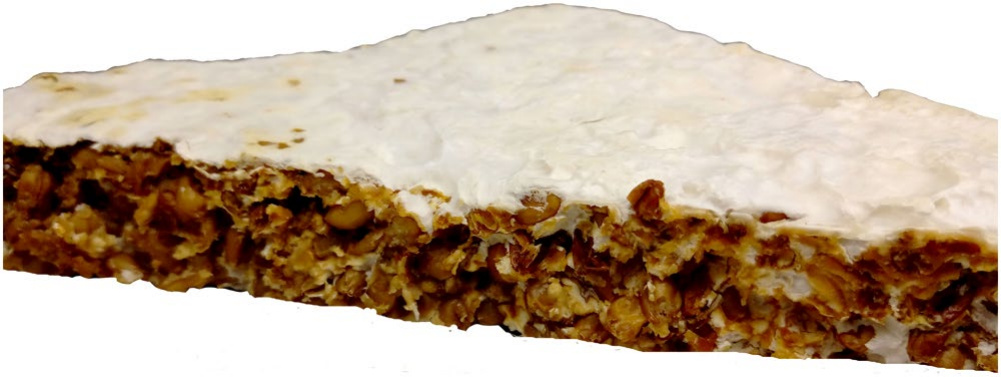
Cross section of the mycelium-wheat composite showing the differences in hyphal density at the surface and the core.

**Figure 8:**
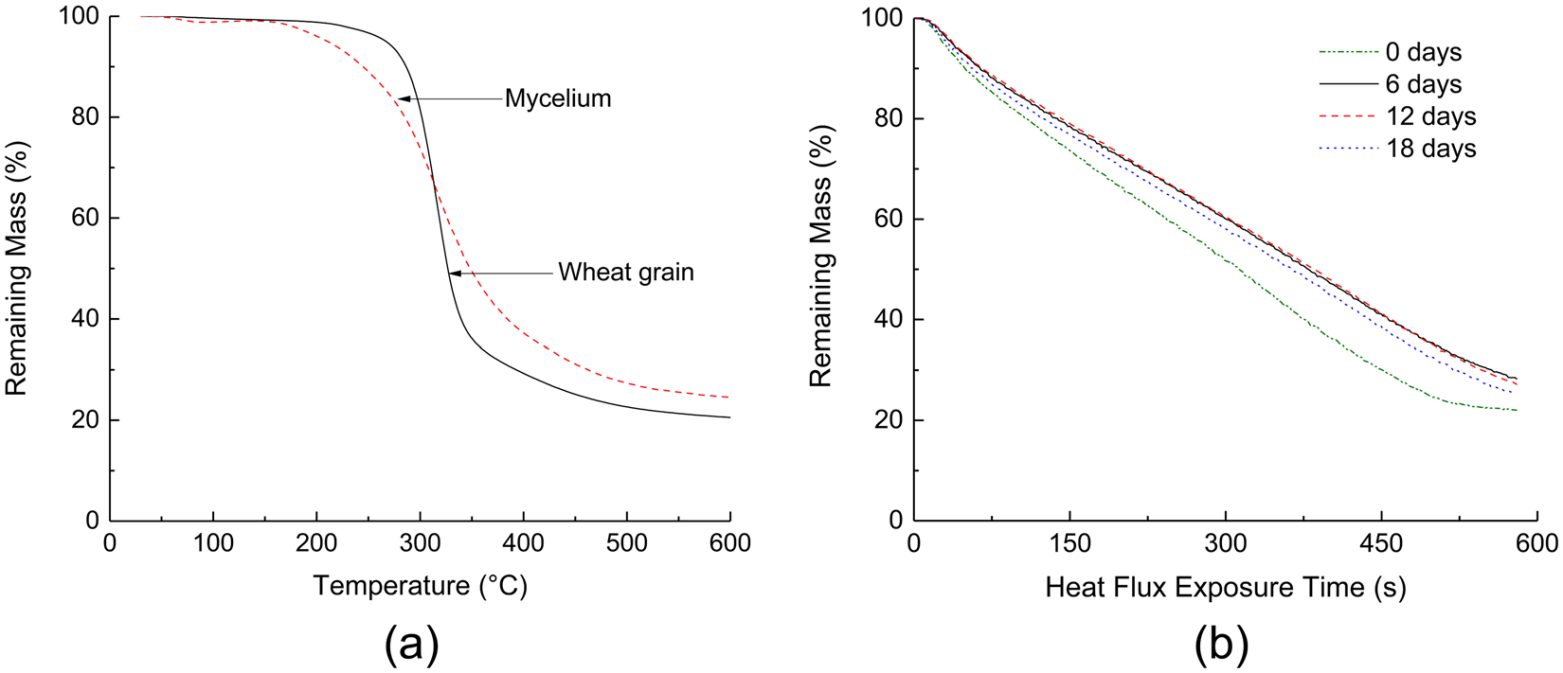
(**a**) TGA – mass loss curves for mycelium and wheat grain tested at 30 °C/min under nitrogen and (**b**) mass loss data for composites grown for 0, 6, 12 and 18 d obtained from cone calorimetry.

The lower PHRR values for the 6, 12 and 18-d samples can be attributed to the formation and growth of a mycelium rich surface layer between 0.9 to 1.5 mm thick depending on growth time (Fig. 7). The hyphal density in the core was significantly lower than at the surface, with negligible differences observed as a function of growth periods. The lower hyphal densities in the core can be attributed to the reduced oxygen diffusion into the bulk of the composite, an essential ingredient for mycelial growth. TGA performed on mycelium and wheat grain (Fig. 8a) showed that mycelium starts to decompose at relatively lower temperatures compared to the wheat grain. However, mycelium is a relatively higher charring material with approximately 23 wt% remaining at 600 °C compared to 19 wt% for wheat grains at the same temperature. The relatively higher mass retention for the 6, 12 and 18-d samples was also confirmed by the cone calorimetry results and is shown in Fig. 8b. The presence of a surface char layer reduces the heat release by acting as a thermal insulator and by limiting the supply of combustible gases and oxygen to the flame front. Following the initial peak, the HRR drops rapidly due to the emergence of a temporary thermally-protective carbonaceous char layer resulting from the thermal degradation of mycelium. However, with continued thermal exposure, the temporary char may degrade thereby exposing the underlying materials and resulting in a second HRR peak event. The absence of mycelium in the 0-d sample meant that the flaming combustion process was uninhibited resulting in a relatively higher second peak HRR value in comparison to the mycelium-coated samples.

**Figure 9:**
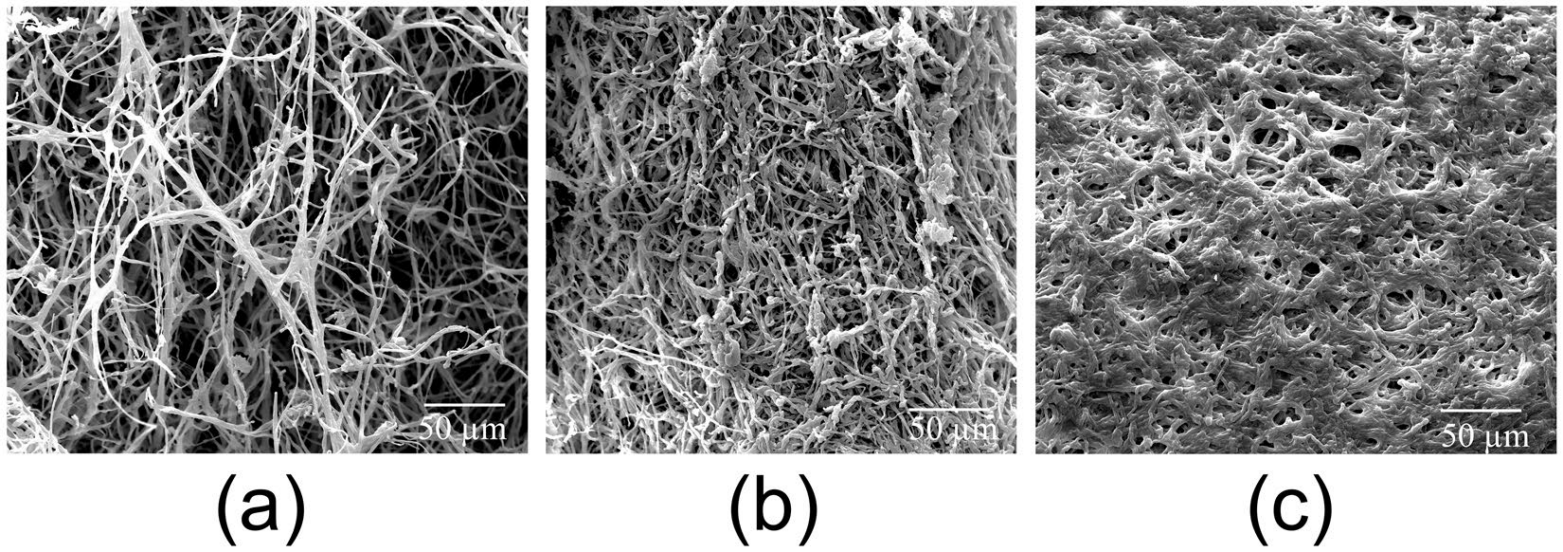
SEM images of mycelium surface layer on composites grown for (**a**) 6 d (**b**) 12 d and (**c**) 18 d.

The fire reaction properties of mycelium composites grown for 6, 12 and 18 d were similar. To elucidate why these composites would achieve the same fire retardation effect despite different growth times, the surface morphologies of the samples were investigated using SEM. As shown in Fig. 9, mycelium composites show a significant increase in the surface hyphal density with increasing growth time. In addition, an increase in the thickness of the surface layer was also observed. The average thickness was measured using an optical microscope and found to be 0.9 ± 0.1, 1.2 ± 0.2 and 1.5 ± 0.2 mm for the 6, 12 and 18-d samples, respectively. However, the increase in thickness of the mycelium coating was not sufficient to cause significant variation in the fire reaction properties of the composite. This observation might point to the existence of a low threshold mycelial density required to enable effective fire retardation in mycelial composites.

## Conclusion

The thermal degradation and fire safety of mycelium and mycelium-wheat grain composites have been characterised using various experimental techniques. Thermogravimetric analysis revealed that the growth time has no discernible effect on the thermal degradation characteristics of mycelium. FTIR and GCMS analysis have identified the complex thermal degradation patterns accompanied by the release of multiple flammable and non-flammable gaseous products. The fibrous structure of mycelium is retained following pyrolysis, albeit with a reduction in its diameter. The fire reaction properties of mycelium have found to be superior to other competing thermoplastic polymers (PMMA and PLA) due to its tendency to form relatively higher char yields. The presence of mycelium is responsible for an improvement in the fire reaction properties of wheat grains. However, beyond 6 d, the growth time has been found to have no significant effect on the fire reaction properties of mycelium-wheat grain composites. Mycelium has been found to possess certain flame-retardant properties (e.g. high char residue and release of water vapour) and could be used as an economical, sustainable and fire-safer alternative to synthetic polymers for binding matrices.

## Electronic supplementary material

Supplementary Material

## Data Availability

The datasets generated during and/or analysed during the current study are available from the corresponding author on reasonable request.
