# Supplementary material for: Thermal Degradation and Fire Properties of Fungal Mycelium and Mycelium - Biomass Composite Materials
Source: Sci Rep. 2018 Dec 4;8:17583. doi: 10.1038/s41598-018-36032-9 (PMC6279784; doi:10.1038/s41598-018-36032-9)
Supplement: Supplementary file 1 — Supplementary Material [file 41598_2018_36032_MOESM1_ESM.pdf]

Electronic supplementary material for:

Thermal Degradation and Fire Properties of Fungal Mycelium and Mycelium - Biomass Composite Materials

Mitchell Jones[1,2†], Tanmay Bhat[1,3†], Everson Kandare[1], Ananya Thomas[4], Paul Joseph[4], Chaitali Dekiwadia[5], Richard Yuen[6], Sabu John[1], Jun Ma[7], Chun-Hui Wang[3*]

[1] School of Engineering, RMIT University, Melbourne 3000, Australia
[2] Institute of Material Chemistry and Research, University of Vienna, Vienna, 1090, Austria
[3] School of Mechanical and Manufacturing Engineering, University of New South Wales, Sydney 2052, Australia
[4] Institute of Sustainable Industries and Liveable Cities, Victoria University, Melbourne 8001, Australia
[5] RMIT Microscopy and Microanalysis Facility, RMIT University, Melbourne 3001, Australia
[6] Department of Architecture and Civil Engineering, City University of Hong Kong, Kowloon, Hong Kong
[7] School of Engineering, University of South Australia, Mawson Lakes, Adelaide 5095, Australia

List of Figures

Figure S1. X-ray photoelectron spectroscopy (XPS) spectra for a) C, N, O and b) P, S, Si for *T. versicolor* mycelial biomass pre- (red) and post-pyrolysis (black). Note: XPS provides environment sensitive, surface based elemental analysis only. It characterises surface chemistry that may be affected by the atmosphere, pyrolysis based chemical reactions and other factors. The energy-dispersive x-ray spectroscopy (EDS) bulk analysis spectra included in the main article are suggested to provide more reliable elemental analysis information for whole mycelial biomass. This information is provided for reference only.

---

*Corresponding author at: School of Mechanical and Manufacturing Engineering, University of New South Wales, Sydney, Australia.
Email address: chun.h.wang@unsw.edu.au (C.H. Wang).

† These authors contributed equally to this work.

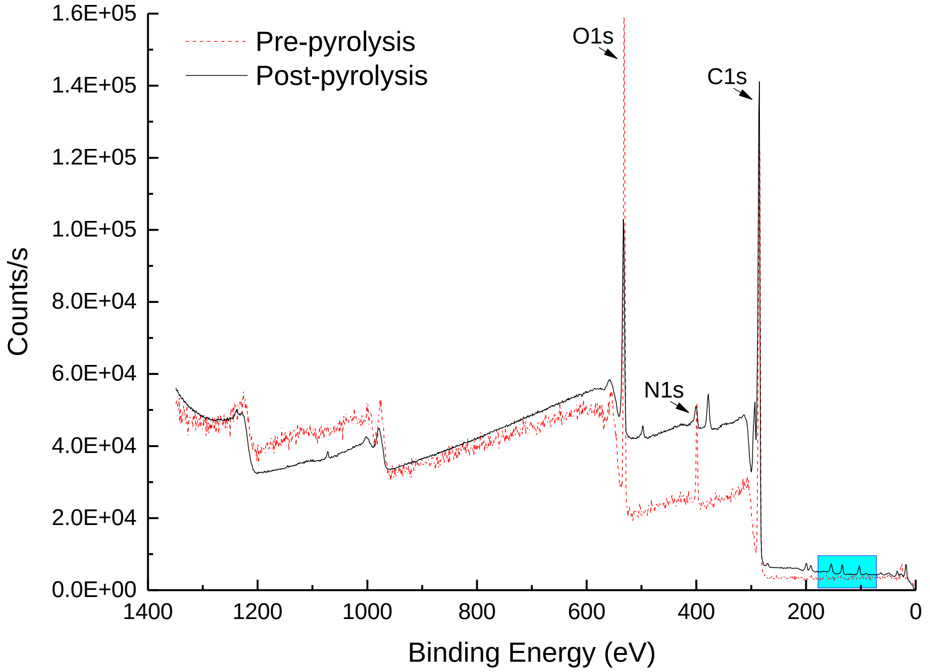

(a)

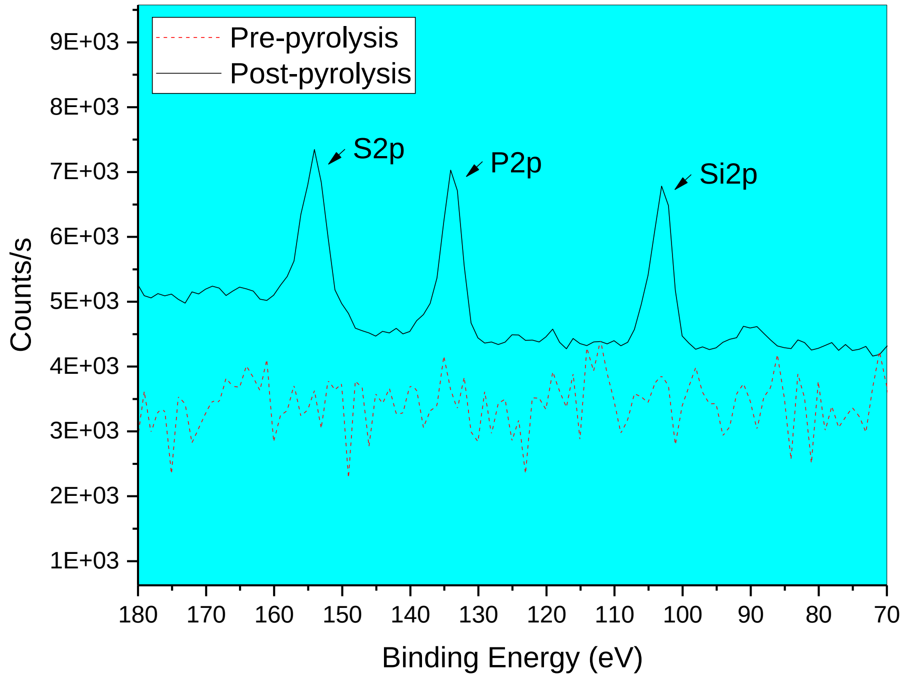

(b)

Figure S1. X-ray photoelectron spectroscopy (XPS) spectra for a) C, N, O and b) P, S, Si for *T. versicolor* mycelial biomass pre- (red) and post-pyrolysis (black). Note: XPS provides environment sensitive, surface based elemental analysis only. It characterises surface chemistry that may be affected by the atmosphere, pyrolysis based chemical reactions and other factors. The energy-dispersive x-ray spectroscopy (EDS) bulk analysis spectra included in the main article are suggested to provide more reliable elemental analysis information for whole mycelial biomass. This information is provided for reference only.
